# Draft Genome Sequences of Mycobacterium tuberculosis Clinical Isolates from the Ural Region of Russia That Carry the *pks15/1* Gene

**DOI:** 10.1128/MRA.01126-19

**Published:** 2019-12-05

**Authors:** Kirill V. Shur, Natalia V. Zakharevich, Natalia I. Akimova, Roman A. Yunes, Svetlana G. Frolova, Dmitry A. Maslov, Valery N. Danilenko

**Affiliations:** a Vavilov Institute of General Genetics Russian Academy of Sciences, Moscow, Russia; University of Maryland School of Medicine

## Abstract

Here, we report the draft genome sequences of 15 Mycobacterium tuberculosis isolates of the Beijing-B0/W-148 sublineage that carry a 7-bp insertion within the *pks15* gene, which leads to the synthesis of Pks15/1 fusion protein. Pks15/1 is involved in phenolglycolipid synthesis and biofilm formation, thus potentially contributing to the B0/W-148 lineage’s enhanced virulence and drug resistance.

## ANNOUNCEMENT

Mycobacterium tuberculosis virulence and pathogenicity are determined by a set of genes which are polymorphic in different phylogenetic lineages (1–3). The M. tuberculosis Beijing genotype has the widest spread; in Russia, it includes the “successful” clone Beijing B0/W-148, which is characterized as multidrug resistant (MDR) and highly virulent (4–6). Therefore, it is very important to analyze lineage-specific virulence and pathogenicity markers. One significant marker is the 7-bp insertion in the *pks15* gene (CCGCGGC) that leads to the synthesis of an active fused Pks15/1 protein (7). Presence of the *pks15/1* gene is correlated with highly virulent and drug-resistant strains of the Beijing genotype (8–10); it plays a significant role in virulence (via phenolic glycolipid synthesis) and may be involved in drug tolerance mediated by biofilm formation (7, 9, 11). Studying the genetic variability of virulence factors such as *pks15/1* is essential for understanding the M. tuberculosis evolutionary process, including the evolution of host-pathogen interaction mechanisms and drug resistance. Thus, we provide the whole-genome sequencing data of 15 strains from a collection of 100 MDR M. tuberculosis Beijing-B0/W-148 strains (12) that carry a copy of the fused *pks15/1* gene.

M. tuberculosis was cultured in Middlebrook 7H9 medium with the addition of oleic acid-albumin-dextrose-catalase (OADC; HiMedia, India) at 37°C for 4 weeks. Genomic DNA was isolated and purified by phenol-chloroform/isoamyl alcohol extraction after enzymatic cell lysis, as described by Belisle et al. (13). The quality of DNA was checked using gel electrophoresis and a Bioanalyzer 2100 instrument (Agilent Technologies, USA). Genomic DNA libraries were prepared using the NEBNext Ultra II DNA library prep kit for Illumina (New England Biolabs, USA). The raw sequencing data were obtained using the HiSeq 2500 platform (Illumina, USA) in rapid run mode with a HiSeq Rapid sequencing by synthesis (SBS) kit v2 (2 ×100 bp; Illumina). The quality check of the reads was done using FastQC v.0.11.7 (14). Illumina reads were *de novo* assembled using SPAdes v.3.12.0 (15) with the -careful flag and k-mers of 21, 33, 55, and 77, while assembly metrics were calculated with QUAST v.5.0.2 with default parameters (16). Automatic functional annotation results were obtained using the NCBI Prokaryotic Genome Annotation Pipeline. Characteristics of the sequenced genomes are listed in Table 1.

**TABLE 1:** Characteristics of 15 M. tuberculosis genome assemblies with the *pks15/1* gene

Isolate	WGS (GenBank) accession no.	SRA accession no.	Genome size (bp)	GC content (%)	Coverage (×)	No. of contigs	Total no. of CDSs^*a*^
EKB1	VOVK00000000	SRR8327216	4,366,764	65.2	99	120	139
EKB3	VOVL00000000	SRR8327860	4,366,227	64.5	99	129	141
EKB10	VOVM00000000	SRR8442761	4,372,733	65.5	100	143	139
EKB12	VOVN00000000	SRR8335026	4,366,328	65.6	99	113	139
EKB23	VOVO00000000	SRR8347538	4,365,633	65.5	99	105	141
EKB28	VOVP00000000	SRR8351921	4,362,713	65.5	99	95	138
EKB29	VOVQ00000000	SRR8352462	4,366,364	65.1	100	129	143
EKB35	VOVR00000000	SRR8353483	4,365,172	65.4	99	106	143
EKB37	VOVS00000000	SRR8354336	4,364,827	65.4	99	108	136
EKB40	VOVT00000000	SRR8354717	4,359,315	65.4	99	103	145
EKB46	VOVU00000000	SRR8357446	4,358,597	65.4	99	100	135
EKB59	VOVV00000000	SRR8365894	4,367,947	65.3	99	121	134
EKB75	VOVW00000000	SRR8369894	4,361,209	64.3	99	121	143
EKB77	VOVX00000000	SRR8370130	4,361,099	65.0	99	123	144
EKB84	VOVY00000000	SRR8379901	4,360,816	65.0	99	132	144

a CDS, coding sequence.

### Data availability.

The whole-genome shotgun (WGS) assemblies described here have been deposited in NCBI GenBank. The versions described in this paper are the first versions. The read archives have been deposited in NCBI SRA. The WGS (GenBank) and SRA accession numbers are listed in Table 1. All of the data are part of BioProject identifier (ID) PRJNA509547.

## References

[1] Prozorov AA, Fedorova IA, Bekker OB, Danilenko VN 2014 The virulence factors of *Mycobacterium tuberculosis*: genetic control, new conceptions. Russ J Genet 50:775–797. doi:10.1134/S1022795414080055. 25731019

[2] Forrellad MA, Klepp LI, Gioffré A, Sabio y, García J, Morbidoni HR, de la Paz Santangelo M, Cataldi AA, Bigi F, Gioffre A, Sabio y Garcia J, Morbidoni HR, de la Paz Santangelo M, Cataldi AA, Bigi F, Gioffré A, Sabio y, García J, Morbidoni HR, de la Paz Santangelo M, Cataldi AA, Bigi F 2013 Virulence factors of the *Mycobacterium tuberculosis* complex. Virulence 4:3–66. doi:10.4161/viru.22329. 23076359 PMC3544749

[3] Danilenko VN, Zaychikova MV, Dyakov IN, Shur KV, Maslov DA 2018 *Mycobacterium tuberculosis*: drug resistance, virulence and possible solutions. Bull Russ State Med Univ 3:5–12.

[4] Hanekom M, Gey Van Pittius NC, McEvoy C, Victor TC, Van Helden PD, Warren RM 2011 *Mycobacterium tuberculosis* Beijing genotype: a template for success. Tuberculosis 91:510. doi:10.1016/j.tube.2011.07.005. 21835699

[5] Mokrousov I, Narvskaya O, Vyazovaya A, Otten T, Jiao WW, Gomes LL, Suffys PN, Shen AD, Vishnevsky B 2012 Russian “successful” clone B0/W148 of *Mycobacterium tuberculosis* Beijing genotype: a multiplex PCR assay for rapid detection and global screening. J Clin Microbiol 50:3757–3759. doi:10.1128/JCM.02001-12. 22933595 PMC3486266

[6] Mokrousov I, Narvskaya O, Vyazovaya A, Millet J, Otten T, Vishnevsky B, Rastogi N 2008 *Mycobacterium tuberculosis* Beijing genotype in Russia: in search of informative variable-number tandem-repeat loci. J Clin Microbiol 46:3576–3584. doi:10.1128/JCM.00414-08. 18753356 PMC2576596

[7] Constant P, Perez E, Malaga W, Lanéelle MA, Saurel O, Daffé M, Guilhot C 2002 Role of the *pks15/1* gene in the biosynthesis of phenolglycolipids in the *Mycobacterium tuberculosis* complex: evidence that all strains synthesize glycosylated *p*-hydroxybenzoic methyl esters and that strains devoid of phenolglycolipids harbor a frameshift. J Biol Chem 277:38148–38158. doi:10.1074/jbc.M206538200. 12138124

[8] Mikheecheva NE, Zaychikova MV, Melerzanov AV, Danilenko VN 2017 A nonsynonymous SNP catalog of mycobacterium tuberculosis virulence genes and its use for detecting new potentially virulent sublineages. Genome Biol Evol 9:887–899. doi:10.1093/gbe/evx053. 28338924 PMC5381574

[9] Zenteno-Cuevas R, Hernandez-Morales RJ, Pérez-Navarro LM, Muñiz-Salazar R, Santiago-García J 2016 A rapid PCR assay to characterize the intact *pks15/1* gene, a virulence marker in *Mycobacterium tuberculosis*. J Microbiol Methods 121:33–35. doi:10.1016/j.mimet.2015.12.006. 26672684

[10] Krishnan N, Malaga W, Constant P, Caws M, Thi Hoang Chau T, Salmons J, Thi Ngoc Lan N, Bang ND, Daffé M, Young DB, Robertson BD, Guilhot C, Thwaites GE 2011 *Mycobacterium tuberculosis* lineage influences innate immune response and virulence and is associated with distinct cell envelope lipid profiles. PLoS One 6:e23870. doi:10.1371/journal.pone.0023870. 21931620 PMC3169546

[11] Pang JM, Layre E, Sweet L, Sherrid A, Moody DB, Ojha A, Sherman DR 2012 The polyketide pks1 contributes to biofilm formation in *Mycobacterium tuberculosis*. J Bacteriol 194:715–721. doi:10.1128/JB.06304-11. 22123254 PMC3264095

[12] Shur KV, Umpeleva TV, Bekker OB, Maslov DA, Zaychikova MV, Vakhrusheva DV, Danilenko VN 2018 Compilation of the *Mycobacterium tuberculosis* Beijing-b0 lineage sample and identifying predictors of immune dysfunction in source patients. Bull Russ State Med Univ 3:23–28.

[13] Belisle JT, Mahaffey SB, Hill PJ 2010 Isolation of *Mycobacterium* species genomic DNA, p 1–12. *In* Parish T, Brown AC (ed), Mycobacteria protocols. Humana Press, Totowa, NJ. 10.1007/978-1-59745-207-6_1 20560080

[14] Andrews S 2015 FastQC a quality control tool for high throughput sequence data. Babraham Institute, Cambridge, UK https://www.bioinformatics.babraham.ac.uk/projects/fastqc/.

[15] Nurk S, Bankevich A, Antipov D, Gurevich AA, Korobeynikov A, Lapidus A, Prjibelski AD, Pyshkin A, Sirotkin A, Sirotkin Y, Stepanauskas R, Clingenpeel SR, Woyke T, McLean JS, Lasken R, Tesler G, Alekseyev MA, Pevzner PA 2013 Assembling single-cell genomes and mini-metagenomes from chimeric MDA products. J Comput Biol 20:714–737. doi:10.1089/cmb.2013.0084. 24093227 PMC3791033

[16] Gurevich A, Saveliev V, Vyahhi N, Tesler G 2013 QUAST: Quality assessment tool for genome assemblies. Bioinformatics 29:1072. doi:10.1093/bioinformatics/btt086. 23422339 PMC3624806

